# Deep learning to predict long-term mortality in patients requiring 7 days of mechanical ventilation

**DOI:** 10.1371/journal.pone.0253443

**Published:** 2021-06-29

**Authors:** Naomi George, Edward Moseley, Rene Eber, Jennifer Siu, Mathew Samuel, Jonathan Yam, Kexin Huang, Leo Anthony Celi, Charlotta Lindvall

**Affiliations:** 1 Department of Emergency Medicine, Division of Critical Care, University of New Mexico Health Science Center, Albuquerque, New Mexico, United States of America; 2 Harvard T.H. Chan School of Public Health, Boston, Massachusetts, United States of America; 3 Department of Psychosocial Oncology and Palliative Care, Dana-Farber Cancer Institute, Boston, Massachusetts, United States of America; 4 Massachusetts Institute of Technology, Cambridge, Massachusetts, United States of America; 5 Université de Montpellier, Montpellier, France; 6 Department of Otolaryngology, Division of Head & Neck Surgery, University of Toronto, Toronto, Canada; 7 Department of Medicine, Beth Israel Deaconess Medical Center, Boston, Massachusetts, United States of America; 8 Department of Medicine, Brigham and Women’s Hospital, Boston, Massachusetts, United States of America; National Yang-Ming University, TAIWAN

## Abstract

**Background:**

Among patients with acute respiratory failure requiring prolonged mechanical ventilation, tracheostomies are typically placed after approximately 7 to 10 days. Yet half of patients admitted to the intensive care unit receiving tracheostomy will die within a year, often within three months. Existing mortality prediction models for prolonged mechanical ventilation, such as the ProVent Score, have poor sensitivity and are not applied until after 14 days of mechanical ventilation. We developed a model to predict 3-month mortality in patients requiring more than 7 days of mechanical ventilation using deep learning techniques and compared this to existing mortality models.

**Methods:**

Retrospective cohort study. Setting: The Medical Information Mart for Intensive Care III Database. Patients: All adults requiring ≥ 7 days of mechanical ventilation. Measurements: A neural network model for 3-month mortality was created using process-of-care variables, including demographic, physiologic and clinical data. The area under the receiver operator curve (AUROC) was compared to the ProVent model at predicting 3 and 12-month mortality. Shapley values were used to identify the variables with the greatest contributions to the model.

**Results:**

There were 4,334 encounters divided into a development cohort (n = 3467) and a testing cohort (n = 867). The final deep learning model included 250 variables and had an AUROC of 0.74 for predicting 3-month mortality at day 7 of mechanical ventilation versus 0.59 for the ProVent model. Older age and elevated Simplified Acute Physiology Score II (SAPS II) Score on intensive care unit admission had the largest contribution to predicting mortality.

**Discussion:**

We developed a deep learning prediction model for 3-month mortality among patients requiring ≥ 7 days of mechanical ventilation using a neural network approach utilizing readily available clinical variables. The model outperforms the ProVent model for predicting mortality among patients requiring ≥ 7 days of mechanical ventilation. This model requires external validation.

## Introduction

Nearly 70% of older adults report that they prioritize quality of life over longevity [1]. Many would prefer death over prolonged survival dependent on mechanical ventilation (MV) [2–5]. Yet, increasingly older adults with acute respiratory failure are treated with MV [6, 7]. By 2020, more than half of the estimated 600,000 critically ill patients treated with MV will be older adults (age ≥ 65 years), and approximately 20% will subsequently undergo tracheostomy [8–13], making tracheostomy one of the most common elective procedure in the intensive care unit (ICU) [14]. Outcomes among older adults who receive tracheostomy are poor; by 12-months 60–70% will have died, and fewer than 10% will have achieved functional independence [10, 12, 13, 15–18]. Identifying the subset of patients who are likely to benefit from tracheostomy, and those who will not, continues to pose a substantial challenge to clinicians [19].

For patients with acute respiratory failure, MV is initially delivered via an endotracheal tube which is passed through the oral cavity. If the respiratory function does not improve after a period of 7–21 days, then the endotracheal tube is often replaced [surgically or percutaneously] with a tracheostomy tube [20–24]. Among patients who survive their acute illness and regain good function, tracheostomy can increase comfort, decrease delirium during MV, and facilitate faster recovery from MV [20–25]. Paradoxically, among the many patients who ultimately do not survive, tracheostomy may serve only to prolong the dying process [15–17, 26]. The toll falls particularly hard on older adults, many of whom will suffer from high rates of distressing symptoms associated with chronic critical illness for weeks or months after tracheostomy but prior to death. Others risk becoming trapped in a state of chronic critical illness, enduring a prolonged but often dismal survival [15–17, 26]. Moreover, healthcare costs and resource utilization associated with the care of older adults who receive tracheostomy are staggering, and do not meet standard thresholds of acceptability for cost effectiveness [27]. Unfortunately, patient’s surrogate decision-makers frequently receive little information regarding the probability of long-term survival and good functional outcome, and often have unrealistic expectations regarding survival [12, 28]. This is due in part clinicians lack of awareness of and comfort with expected prognosis [29–31].

Existing general ICU mortality prediction models, such as the Acute Physiology and Chronic Health Evaluation (APACHE) score, were developed to predict in-hospital mortality and perform poorly in predicting long term survival of patients requiring prolonged mechanical ventilation [32, 33]. Clinical tools such as the ProVent Score can be used to predict 1-year mortality after 14 or 21 days of MV. Such tools were developed with the intention of informing prognosis in decision-making conversations around tracheostomy [34–38]. However, multiple randomized trials have demonstrated that ‘early’ tracheostomy placement (< 10 days after initiation of MV) results in decreased need for sedatives, fewer days of MV, decreased ICU stay, and may be associated with decreased long-term mortality as compared to ‘late’ (>10 days) tracheostomy placement [22, 39]. Thus, more than 50% of tracheostomies are now placed early (<10–14 days) [39], at which time the validity of the ProVent Score is unknown. Among patients with poor prognosis, enduring 14–21 days of ICU care prior to prognostication may be unnecessarily burdensome.

To address this gap, we sought to develop a mortality prediction model to enhance decision making around tracheostomy. Our objective was to develop and validate deep learning model to predict 3- and 12-month mortality among patients requiring more than 7 days of mechanical ventilation for acute respiratory failure.

## Methods

### Design, setting, and population

Data were obtained from the Medical Information Mart for Intensive Care III (MIMIC-III) database. MIMIC-III contains records of 61,051 ICU admissions at Beth Israel Deaconess Medical Center in Boston, Massachusetts from June 2001, through October, 2012 [40]. Inclusion criteria included patients over the age of 18 years admitted to the neurological, trauma, surgical, cardiac, or medical ICU and who were treated with MV for ≥ 7 days. Patients were excluded if they had a primary hospital diagnosis of head and neck cancer requiring surgical intervention in the neck, burns comprising >30% body area, burns involving the head and neck, or acute neuromuscular disorders (e.g. Guillain Barre) (**S1 Table**). The data in MIMIC-III has been previously de-identified, and the institutional review boards of the Massachusetts Institute of Technology (No. 0403000206) and Beth Israel Deaconess Medical Center (2001-P-001699/14) both approved the use of the database for research. The Guidelines for Developing and Reporting Machine Learning Predictive Models in Biomedical Research were followed throughout this project [41].

### Feature selection and data processing

**Fig 1:**
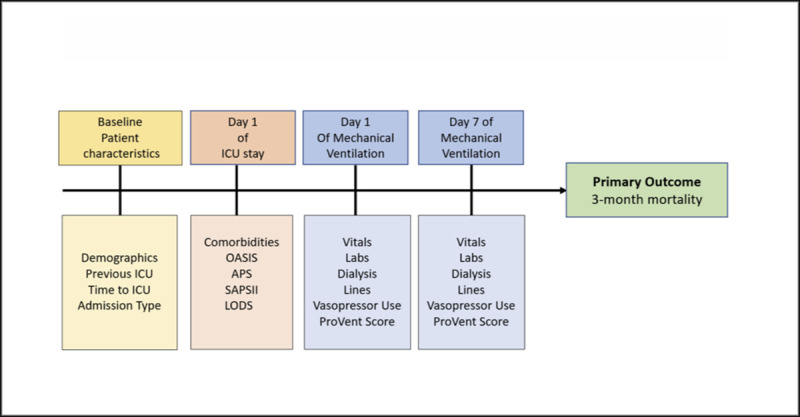
Variable selection for machine learning neural network prediction model.

Processes-of-care variables selected for inclusion in the model were reviewed by meidcal clinicians (NG, CL, JS) and from existing ICU mortality prediction literature and included all the variables from the ProVent Model **(Fig 1)**. Demographic variables were carefully selected to avoid overt reinforcement of health disparities. Thus race, ethnicity, insurance status, zip code were all excluded from the model.

The extracted data contained both static information, (e.g. age, sex, duration of hospitalization prior to ICU admission, reason for admission, site of admission, and International Classification of Diseases, 9th Revision codes), as well as temporal and dynamic data (e.g. time-stamped laboratory values, vital signs, medication administrations). Feature engineering was performed on continuous variables (e.g. maximum value, minimum value, and mean value). Only variables obtained from the first 7 days of MV were included. Continuous variables were standardized to a mean of zero and scaled to unit variance. Additionally, five severity of illness scores were included as predictors: the Oxford Acute Severity of Illness Score (OASIS), the Simplified Acute Physiology Score (SAPS), the Simplified Acute Physiology Score Version II (SAPSII), the Acute Physiology Score III (APSIII), and the Logistic Organ Dysfunction System (LODS) [42–47]. The Elixhauser van Walraven comorbidity score and its 30 component items were also included [48]. Mortality data was obtained from the Social Security Death Index.

### Model building and validation

The dataset was randomly divided into a training (80%) and a testing set (20%). A multi-layer feedforward neural network was created. Neural networks approximate the best separating function for labeled input data and can learn any arbitrary, complex functions. They do not use weight-sharing across layers; information flows in one-direction, from the input layers, through intermediate hidden layers to the output layer. A sigmoid activation function was used in the output layer, with which the output range was interpreted as a prediction probability of the primary outcome, 3-month mortality (**S1 Fig**). For better generalization capabilities, we utilized dropout and L1-L2 regularization in each hidden layer. These techniques prevent complex co-adaptations on training data, preventing overfitting. For missing or outlier values of continuous data, normal values were imputed. Individual outliers were reviewed and discarded if deemed erroneous.

### Statistical analysis and outcomes

The primary outcome was 3-month mortality. The final model chosen was based on the highest calibration determined by area under the receiver operator curve (AUROC) in the testing set. The secondary outcome was 12-month mortality. Sensitivity, specificity, AUROC, accuracy and F1 score are also reported. Shapley values, which reveal the marginal contribution of the individual variables across permutations, were reported to facilitate understanding of the model [49]. The neural network models’ predictive ability was compared to the ProVent model. The ProVent model was developed to predict mortality among patients requiring MV for 14 days or more, and has been validated in several studies since its publication [34–37, 50]. We calculated the performance of the ProVent score using the component variables as reported in the original manuscript. In addition, in order to give the logistic regression model the ‘best chance’ to compete with the deep learning model we also built ‘extended’ logistic regression models using the ProVent variables as well as each of the other variables available to the deep learning model using 10 fold cross-validation (*extended LR*) and least absolute shrinkage and selection operator (*extended LR LASSO*) techniques. The AUROC for was calculated for ProVent logistic regression based on values at 7 days of MV. Statistical analysis and model building was performed using Python v3.6 [51].

## Results

**Fig 2:**
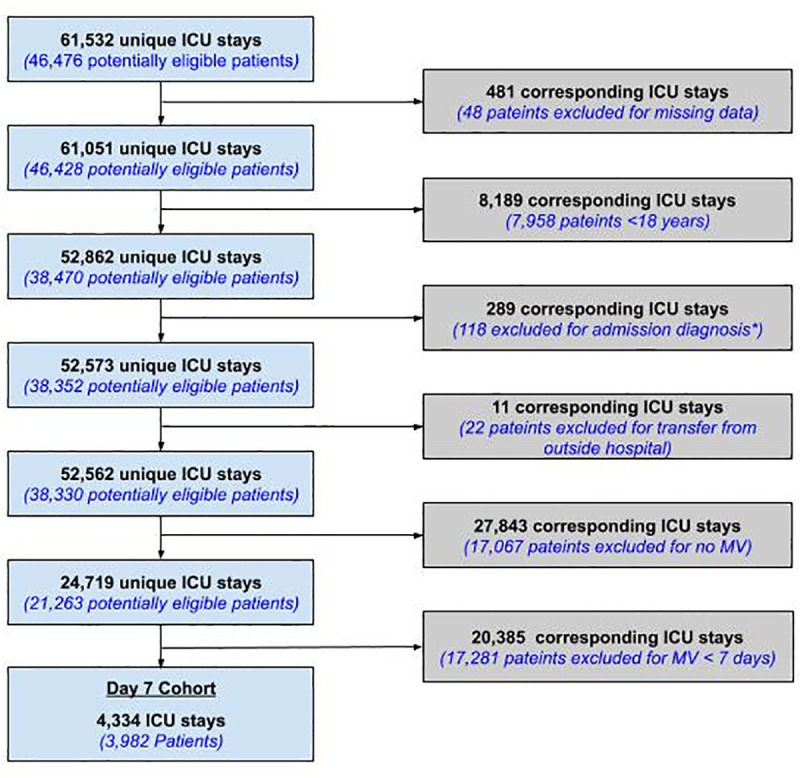
Flow diagram.

A total of 61,532 unique ICU stays were identified within the MIMIC database. Of those, there were 4,334 unique ICU stays representing 3,982 unique ICU patients receiving ≥ 7 days of MV and meeting the inclusion and exclusion criteria. Patient selection is outline in **Fig 2**. Eighty percent (n = 3,467) of this study sample were used as the initial training data set and the remaining 20% (n = 867) were used to test the prediction models.

Of the 3,982 patients that met inclusion criteria 59.6% were alive at 3 months. Baseline patient characteristics are summarized in **Table 1**. The median age was 65.9 years (interquartile range (IQR) 52.7, 76.8). The majority of patients (70.9%) were white, admitted to the ICU from the emergency room 49.4%. Median ICU length of stay was 15.7 days (IQR of 10.8, 22.5). The development and testing cohorts did not significantly differ in terms of illness severity or baseline comorbidities with the exception of baseline chronic obstructive pulmonary disease and metastatic cancer. Laboratory values, vital signs, and clinical interventions were not significantly different between development and testing sets (**S2 Table)**.

**Table 1:** Patient characteristics.

	Training n = 3,467	Testing n = 867	p-value
**Sex**			
Female, n (%)	1494 (43.1)	361 (41.6)	0.462
**Age (years)**			
Median (IQR)	65.8 (52.9, 76.8)	65.9 (52.2, 76.9)	0.694
**Race/Ethnicity n (%)**			
Asian	77 (2.2)	14 (1.6)	0.661
Non-Hispanic Black	277 (8.0)	73 (8.4)	
Hispanic	91 (2.6)	19 (2.2)	
Unknown/Others	562 (16.2)	150 (17.3)	
Non-Hispanic White	2460 (71.0)	611 (70.5)	
**Source Location of ICU Admission, n (%)**			
Emergency Department	428 (49.4)	1724 (49.7)	0.397
Office Referral	218 (25.1)	928 (26.8)	
Transfer from Hospital or SNF	221 (25.5)	815 (23.5)	
**First Care Unit, n (%)**			
Coronary Care Unit	355 (10.2)	94 (10.8)	0.374
Cardiac Surgery Recovery Unit	436 (12.6)	108 (12.5)	
Medical Intensive Care Unit	1382 (39.9)	372 (42.9)	
Surgical Intensive Care Unit	706 (20.4)	162 (18.7)	
Trauma/Surgical Intensive Care Unit	588 (17.0)	131 (15.1)	
**Illness Severity on ICU Admission (median, IQR)**			
SOFA Score	6.0 (4.0, 9.0)	6.0 (4.0, 9.0)	0.43
LODS	6.0 (4.0, 8.0)	6.0 (3.0, 8.0)	0.736
OASIS	38.0 (32.0, 44.0)	38.0 (33.0, 44.0)	0.286
SAPS II	42.0 (33.0, 52.5)	43.0 (34,0, 54.0)	0.227
**Elixhauser Comorbidities, n (%)**			
Elixhauser Score, (median, IQR)	4.0 (2.0, 5.0)	4.0 (2.0, 5.0)	0.41
Congestive Heart Failure	1227 (35.4)	325 (37.5)	0.267
Chronic Pulmonary Disease	881 (25.4)	239 (27.6)	0.21
Liver disease	562 (16.2)	142 (16.4)	0.945
Renal Failure	520 (15.0)	117 (13.5)	0.287
Metastatic Cancer	131 (3.8)	33 (3.8)	1.000
**Mortality, n%**			
Time to death, days (median, IQR)	55.1 (18.2, 314.0)	52.2 (18.2, 209.9)	0.309
3-month Mortality	1390 (40.1)	360 (41.5)	0.466
12-month Mortality	1714 (49.4)	428 (49.4)	1.000
**Length of Stay (LOS), median (IQR)**			
Hospital LOS	22.57 (15.7, 32.3)	22.32 (15.7, 31.6)	0.713
ICU Length of Stay	15.69 (11.6, 23.1)	15.78 (11.5, 23.8)	0.863
**Hospital Disposition, n (%)**			
Died	979 (28.2)	250 (28.8)	0.651
Home / Home health care	396 (11.4)	111 (12.8)	
Hospice	26 (0.7)	9 (1.0)	
Long Term Acute Care	468 (13.5)	106 (12.2)	
Short Term Hospital/other	40 (1.2)	12 (1.4)	
Subacute Nursing Facility (SNF)	1558 (44.9)	379 (43.7)	

**Fig 3:**
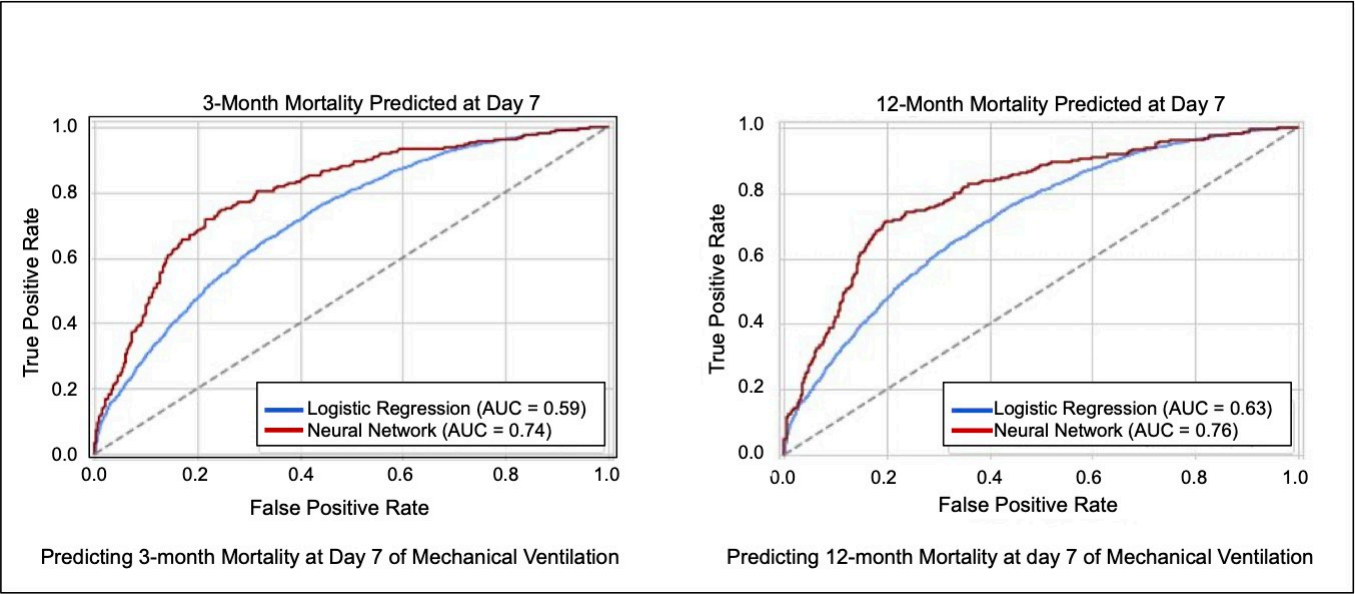
Predicting 3- and 12-month mortality at day 7 of mechanical ventilation.

The final deep learning model included 250 variables. The performance of the neural network and the ProVent Model for prediction of 3- and 12-month mortality at day is shown in **Table 2**. The performance of the model for predicting 3-month and 12-month mortality status is shown in **Fig 3**. The day 7 neural network model had an AUROC of 0.74 for 3-month mortality and 0.76 for 12-month mortality, versus 0.59 and 0.63 for the ProVent model, respectively. Of note, in our cohort, the ProVent Model performed worse when measured at 7 days (AUC 0.59) than in previously published studies. The positive and negative predictive value for the neural network at 3 months was 0.64 and 0.72 respectively, and 0.67 and 0.71 at 12 months. The calibration curve for the testing and training set for both 3-month and 12-month mortality are shown in **S2 Fig**, and the area under the precision recall curves are shown in **S3 Fig**. In addition, comparison of the neural network model with the extended LR and extended LR LASSO models are available in **S3 Table**.

**Table 2:** Deep model performance versus ProVent.

Model	Three-Month Mortality	12-Month Mortality
**Deep Learning at 7 Days**		
Area under ROC	0.74	0.76
Sensitivity	0.58	0.72
Specificity	0.76	0.65
Accuracy	0.69	0.69
F1 Score	0.61	0.69
**ProVent at 7 Days**		
Area under ROC	0.59	0.63
Sensitivity	0.41	0.73
Specificity	0.78	0.53
Accuracy	0.63	0.63
F1 Score	0.47	0.66

**Fig 4:**
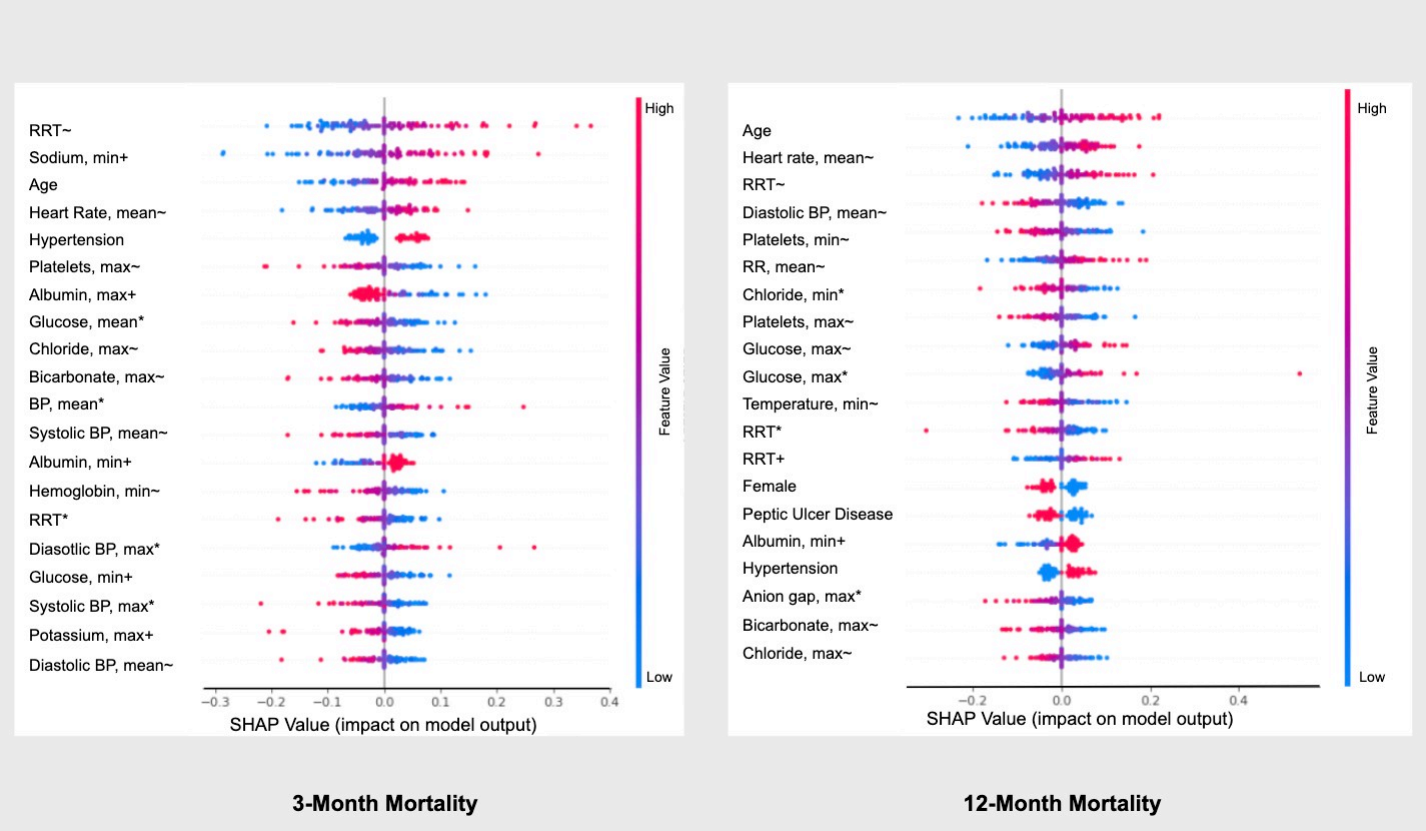
SHAP values for 3 and 12-month mortality. *value on ICU day 1 of ICU; ^+^ value on day 1 mechanical ventilation; ^~^ value on day 7 of mechanical ventilation; RRT: Renal Replacement Therapy; min: minimum value; max: maximum value; BP: blood pressure; RR: respiratory rate.

Analysis of relative variable importance to the model prediction using shapley values showed that increased use of renal replacement therapy on day 7 of MV, elevated sodium levels on day 1 of MV, increased age, and increased heart rate on day 7 of MV had the largest impact on the model’s prediction of 3-month. Similarly, increased age, increased heart rate on day 7 of MV, use of renal replacement therapy on day 7 of MV, and low diastolic blood pressure on day 7 had the greatest impact on the model’s prediction of 12-month mortality **(Fig 4)**.

## Discussion

We demonstrate that a feedforward neural network model, based on clinical variables readily available during routine processes-of-care in the ICU, can accurately predict 3- and 12-month mortality among ICU patients requiring mechanical ventilation for ≥ 7 days. Our model had superior performance at 7 days compared to one of the most commonly used mortality prediction models for mechanically ventilated patients, the ProVent Score, and can be applied earlier in a patients’ ICU course.

Predicting which ICU patients are at high risk for a poor outcome is essential in order to make more informed decisions regarding medical interventions at the end of life, and minimize suffering for patients who are on a dying trajectory [15, 52, 53]. However, mortality risk prediction models among ICU patients are infrequently used to inform clinical decisions [12, 28–31]. This is attributable to several factors. First, the most common ICU mortality prediction models, such as the APACHE, SAPS, LODS, and MPM scores, are derived from data obtained in the first 24 hours of the ICU stay. Yet, many patients and families prefer to attempt a trial of therapy (typically several days in duration), prior to goals of care decision-making, after which predictions gleaned during the first 24 hours may no longer accurately reflect the prognosis. Second, most mortality prediction models, including the SOFA score and APACHE score, are calibrated to predict in-hospital mortality [32, 33, 43]. However, post-discharge prognosis can also contribute to more meaningful discussions with patients.

Among patients surviving ≥ 7 days of mechanical ventilation, one of the most critical decisions clinicians, patients, and families will face is whether or not to undergo tracheostomy. Among patients on mechanical ventilation with a good long-term prognosis, transition to tracheostomy can improve comfort, facilitate early mobility, and enhance recovery [54]. However, for older adult ICU survivors, those requiring ongoing treatment with mechanical ventilation often face a dismal quality of life, dying within a year of tracheostomy placement [55].

For most ICU patients requiring MV the decision of whether to undergo tracheostomy falls to a surrogate decision maker(s), who in turn relies on the ICU clinicians to share information about prognosis and guide expectations. Surrogate decision-makers often seek prognostic disclosures from clinicians [30]. However, studies have shown significant problems with communication of ICU prognosis; information may be subject to bias and is often poorly communicated by clinicians, misunderstood by families, or never disclosed at all [12, 28]. Clinicians themselves have only modestly accurate prognostic ability in terms of mortality–often overestimating the probability of a survival [31, 56]. If surrogate decision-makers of patients with poor prognosis were made aware of risk for poor outcome, it is likely many would not choose tracheostomy and ongoing MV [2, 57].

It is imperative that clinicians have prognostic information about the probability of survival after tracheostomy prior to placement of tracheostomy in order to facilitate decision-making. Among long-term survivors, delay in tracheostomy decision past 10 days may threaten the patient’s ability to maximally benefit from tracheostomy, and increases the duration of burdensome symptoms associated with endotracheal tubes. Among decedents, the delay in tracheostomy decision may mean a missed opportunity for earlier transition to hospice care or natural death with less suffering than results from continued ICU care with mechanical ventilation.

Severity scores like ProVent are based on logistic regression models that assume linear and additive relationship of predictors and outcomes. However, these assumptions may not be valid in the context of critical illness with very complex underlying processes and complicated interactions between patient factors, disease features and treatments administered. Indeed, in our study the ProVent score performed poorly as compared to the machine learning model. This is likely due in part to the fact that the ProVent model was not developed for application at day 7, and also due to the strengths of machine learning. Machine learning methods can offer a more flexible statistical approach and have been shown to outperform conventional logistic regression statistical models for several medical conditions [58, 59]. Interestingly, in our study the LASSO model, which is a hybrid between regression and machine learning techniques, significantly outperformed the ProVent model giving results similar to the neural network.

In terms of accuracy, our model compares similarly to other machine learning mortality prediction models developed for critically ill patients. Dybowski et. al. used a cohort of 258 ICU patients to create an artificial neural network enhanced by generic algorithms achieving an AUC 0.86, however their study was limited to a small subset of patients who either had a systemic inflammatory response syndrome or hemodynamic shock [60]. Pirracchio et al developed a “Super Learner” model using ensemble machine learning technique with multiple learning algorithms to predict in-hospital mortality [58]. Kim et. al compared decision trees, artificial neural networks, and support vector machine models predicting mortality in pediatric ICU patients, achieving an AUC 0.87–0.89, outperforming traditionally used regression models [61].

To the best of our knowledge our model is the first to predict long-term mortality among mechanically ventilated patients ≥ 7 days of MV. Our approach is strengthened in the use multiple time points within a patient’s clinical trajectory (baseline variables collected at ICU admission, then day 1 of MV, and day 7 MV) which allows for flexibility and time-sequence analysis in the event of patient status changes during their clinical course.

There are limitations to this study, and the results should only be interpreted in the context of its study design. All database analyses are susceptible to coding misclassification and bias from error. Further, the data comes from a single institution, whose organization and clinical practice patterns may differ from other institutions, limiting the generalizability of our model. In addition, the data in MIMIC only extends to 2012. Our data was compared to the ProVent prediction model which was optimized for day 14 and 21 of mechanical ventilation. Because we believe day 7 on mechanical ventilation to be more clinically relevant, this comparison may be limited. Nonetheless this study represents an important methodology to build a clinical tool that can provide more detailed insight into the prognosis of patients who may require tracheostomy.

## Conclusion

Here we demonstrate the ability of using a neural network to predict 3- month mortality in patients on mechanical ventilation for more than 7 days. Deep learning prediction models are becoming increasingly important in our data-driven clinical decision-making, especially in the absence of randomized controlled trials. Further optimization of these by external validation, prospective validation using external cohorts, and subgroup analysis within specific populations is integral prior to widespread implementation. Ultimately, integration of deep learning prediction models like ours into electronic health records will provide valuable information to enable providers and patients to more informed decisions.

## Supporting information

S1 Fig Schematic diagram of forward feed neural network model. (TIF)

S2 Fig 3 and 12-month calibration curve. (TIF)

S3 Fig 3- and 12-month area under the precision recall curve. (TIF)

S1 Table Exclusion criteria ICD-9 code. (TIF)

S2 Table Vital signs and laboratory values. (TIF)

S3 Table Deep model performance versus ProVent. (TIF)

## References

[1] Steinhauser KE, Christakis NA, Clipp EC, McNeilly M, McIntyre L, Tulsky JA. Factors considered important at the end of life by patients, family, physicians, and other care providers. JAMA. 2000;284(19):2476–82. doi: 10.1001/jama.284.19.2476 11074777

[2] Heyland DK, Dodek P, Rocker G, Groll D, Gafni A, Pichora D, et al. What matters most in end-of-life care: perceptions of seriously ill patients and their family members. CMAJ Can Med Assoc J J Assoc Medicale Can. 2006;174(5):627–33. doi: 10.1503/cmaj.050626 16505458 PMC1389825

[3] Singer PA, Martin DK, Kelner M. Quality end-of-life care: patients’ perspectives. JAMA. 1999;281(2):163–8. doi: 10.1001/jama.281.2.163 9917120

[4] Chochinov HM, Hack T, Hassard T, Kristjanson LJ, McClement S, Harlos M. Dignity in the terminally ill: a cross-sectional, cohort study. Lancet Lond Engl. 2002;360(9350):2026–30. doi: 10.1016/S0140-6736(02)12022-8 12504398

[5] Steinhauser KE, Clipp EC, McNeilly M, Christakis NA, McIntyre LM, Tulsky JA. In search of a good death: observations of patients, families, and providers. Ann Intern Med. 2000;132(10):825–32. doi: 10.7326/0003-4819-132-10-200005160-00011 10819707

[6] Abe T, Madotto F, Pham T, Nagata I, Uchida M, Tamiya N, et al. Epidemiology and patterns of tracheostomy practice in patients with acute respiratory distress syndrome in ICUs across 50 countries. Crit Care Lond Engl. 2018;22(1):195. doi: 10.1186/s13054-018-2126-6 30115127 PMC6097245

[7] Mehta AB, Syeda SN, Bajpayee L, Cooke CR, Walkey AJ, Wiener RS. Trends in Tracheostomy for Mechanically Ventilated Patients in the United States, 1993–2012. Am J Respir Crit Care Med. 2015;192(4):446–54. doi: 10.1164/rccm.201502-0239OC 25955332 PMC4595669

[8] Zilberberg MD, de Wit M, Pirone JR, Shorr AF. Growth in adult prolonged acute mechanical ventilation: Implications for healthcare delivery. Crit Care Med. 2008;36(5):1451–5. doi: 10.1097/CCM.0b013e3181691a49 18434911

[9] Ely EW, Baker AM, Dunagan DP, Burke HL, Smith AC, Kelly PT, et al. Effect on the duration of mechanical ventilation of identifying patients capable of breathing spontaneously. N Engl J Med. 1996;335(25):1864–9. doi: 10.1056/NEJM199612193352502 8948561

[10] Cox CE, Carson SS, Holmes GM, Howard A, Carey TS. Increase in tracheostomy for prolonged mechanical ventilation in North Carolina, 1993–2002. Crit Care Med. 2004;32(11):2219–26. doi: 10.1097/01.ccm.0000145232.46143.40 15640633

[11] Hsu Chia-Lin, Chen Kuan-Yu, Chang Chia-Hsuin, Jerng Jih-Shuin, Yu Chong-Jen, Yang Pan-Chyr. Timing of tracheostomy as a determinant of weaning success in critically ill patients: a retrospective study. Crit Care. 2004;9(1):R46. doi: 10.1186/cc3018 15693966 PMC1065112

[12] Cox CE, Martinu T, Sathy SJ, Clay AS, Chia J, Gray AL, et al. Expectations and outcomes of prolonged mechanical ventilation. Crit Care Med. 2009;37(11):2888–94. doi: 10.1097/CCM.0b013e3181ab86ed 19770733 PMC2766420

[13] Zilberberg MD, Luippold RS, Sulsky S, Shorr AF. Prolonged acute mechanical ventilation, hospital resource utilization, and mortality in the United States. Crit Care Med. 2008;36(3):724–30. doi: 10.1097/CCM.0B013E31816536F7 18209667

[14] Esteban A, Anzueto A, Alía I, Gordo F, Apezteguía C, Pálizas F, et al. How Is Mechanical Ventilation Employed in the Intensive Care Unit? An International Utilization Review. Am J Respir Crit Care Med. 2000;161(5):1450–8. doi: 10.1164/ajrccm.161.5.9902018 10806138

[15] Unroe M, Kahn JM, Carson SS, Govert JA, Martinu T, Sathy SJ, et al. One-year trajectories of care and resource utilization for recipients of prolonged mechanical ventilation: a cohort study. Ann Intern Med. 2010;153(3):167–75. doi: 10.7326/0003-4819-153-3-201008030-00007 20679561 PMC2941154

[16] Carson SS, Bach PB, Brzozowski L, Leff A. Outcomes after long-term acute care. An analysis of 133 mechanically ventilated patients. Am J Respir Crit Care Med. 1999;159(5 Pt 1):1568–73. doi: 10.1164/ajrccm.159.5.9809002 10228128

[17] Nelson JE, Meier DE, Litke A, Natale DA, Siegel RE, Morrison RS. The symptom burden of chronic critical illness. Crit Care Med. 2004;32(7):1527–34. doi: 10.1097/01.ccm.0000129485.08835.5a 15241097

[18] Fisher M, Ridley S. Uncertainty in end-of-life care and shared decision making. Crit Care Resusc. 2012;14(1):81–7. 22404067

[19] Bice T, Nelson JE, Carson SS. To Trach or Not to Trach: Uncertainty in the Care of the Chronically Critically Ill. Semin Respir Crit Care Med [Internet]. 2015; Available from: doi: 10.1055/s-0035-1564872 26595045 PMC4809243

[20] Mallick A, Bodenham AR. Tracheostomy in critically ill patients. Eur J Anaesthesiol. 2010;27(8):676–82. doi: 10.1097/EJA.0b013e32833b1ba0 20523214

[21] Freeman BD, Morris PE. Tracheostomy practice in adults with acute respiratory failure. Crit Care Med. 2012;40(10):2890–6. doi: 10.1097/CCM.0b013e31825bc948 22824938

[22] Young D, Harrison DA, Cuthbertson BH, Rowan K, for the TracMan Collaborators. Effect of Early vs Late Tracheostomy Placement on Survival in Patients Receiving Mechanical Ventilation: The TracMan Randomized Trial. Surv Anesthesiol. 2014;58(2):65–6. 10.1001/jama.2013.5154 23695482

[23] Andriolo BN, Andriolo RB, Saconato H, Atallah ÁN, Valente O. Early versus late tracheostomy for critically ill patients. Cochrane Database Syst Rev. 2015;1:CD007271. doi: 10.1002/14651858.CD007271.pub3 25581416 PMC6517297

[24] Gomes Silva BN, Andriolo RB, Saconato H, Atallah AN, Valente O. Early versus late tracheostomy for critically ill patients. Cochrane Database Syst Rev. 2012;(3):CD007271. doi: 10.1002/14651858.CD007271.pub2 22419322

[25] Szakmany T., Russell P., Wilkes A.R., Hall J.E. Effect of early tracheostomy on resource utilization and clinical outcomes in critically ill patients: Meta-analysis of randomized controlled trials. Br J Anaesth. 2015;114(3):396–405. doi: 10.1093/bja/aeu440 25534400

[26] Nelson JE, Tandon N, Mercado AF, Camhi SL, Ely EW, Morrison RS. Brain dysfunction: another burden for the chronically critically ill. Arch Intern Med. 2006;166(18):1993–9. doi: 10.1001/archinte.166.18.1993 17030833

[27] Cox CE, Carson SS, Govert JA, Chelluri L, Sanders GD. An economic evaluation of prolonged mechanical ventilation. Crit Care Med. 2007;35(8):1918–27. doi: 10.1097/01.CCM.0000275391.35834.10 17581479 PMC2745076

[28] Nelson JE, Mercado AF, Camhi SL, Tandon N, Wallenstein S, August GI, et al. Communication About Chronic Critical Illness. Arch Intern Med. 2007 Dec 10;167(22):2509–15. doi: 10.1001/archinte.167.22.2509 18071175 PMC3157319

[29] Christakis NA, Iwashyna TJ. Attitude and self-reported practice regarding prognostication in a national sample of internists. Arch Intern Med. 1998 Nov 23;158(21):2389–95. doi: 10.1001/archinte.158.21.2389 9827791

[30] Evans LR, Boyd EA, Malvar G, Apatira L, Luce JM, Lo B, et al. Surrogate decision-makers’ perspectives on discussing prognosis in the face of uncertainty. Am J Respir Crit Care Med. 2009 Jan 1;179(1):48–53. doi: 10.1164/rccm.200806-969OC 18931332 PMC2615661

[31] Meadow W, Pohlman A, Frain L, Ren Y, Kress JP, Teuteberg W, et al. Power and limitations of daily prognostications of death in the medical intensive care unit. Crit Care Med. 2011 Mar;39(3):474–9. doi: 10.1097/CCM.0b013e318205df9b 21150582

[32] Carson SS, Bach PB. Predicting mortality in patients suffering from prolonged critical illness: an assessment of four severity-of-illness measures. Chest. 2001;120(3):928–33. doi: 10.1378/chest.120.3.928 11555531

[33] Angus DC, Clermont G, Kramer DJ, Linde-Zwirble WT, Pinsky MR. Short-term and long-term outcome prediction with the Acute Physiology and Chronic Health Evaluation II system after orthotopic liver transplantation. Crit Care Med. 2000;28(1):150–6 doi: 10.1097/00003246-200001000-00025 10667515

[34] Carson SS, Garrett J, Hanson LC, Lanier J, Govert J, Brake MC, et al. A prognostic model for one-year mortality in patients requiring prolonged mechanical ventilation. Crit Care Med. 2008;36(7):2061–9. doi: 10.1097/CCM.0b013e31817b8925 18552692 PMC2728216

[35] Hough CL, Caldwell ES, Cox CE, Douglas IS, Kahn JM, White DB, et al. Development and Validation of a Mortality Prediction Model for Patients Receiving 14 Days of Mechanical Ventilation. Crit Care Med. 2015 Nov;43(11):2339–45. doi: 10.1097/CCM.0000000000001205 26247337 PMC4788381

[36] Kim W-Y, Jo E-J, Eom JS, Mok J, Kim M-H, Kim KU, et al. Validation of the Prognosis for Prolonged Ventilation (ProVent) score in patients receiving 14 days of mechanical ventilation. J Crit Care. 2018;44:249–54. doi: 10.1016/j.jcrc.2017.11.029 29202432

[37] Park YR, Lee JS, Kim HJ, Hong S-B, Lim C-M, Koh Y, et al. Modification of the prolonged mechanical ventilation prognostic model score to predict short-term and 1-year mortalities. Respirol Carlton Vic. 2019;24(2):179–85. doi: 10.1111/resp.13400 30223306

[38] Carson SS, Kahn JM, Hough CL, Seeley EJ, White DB, Douglas IS, et al. A multicenter mortality prediction model for patients receiving prolonged mechanical ventilation. Crit Care Med. 2012 Apr;40(4):1171–6. doi: 10.1097/CCM.0b013e3182387d43 22080643 PMC3395423

[39] Hosokawa K, Nishimura M, Egi M, Vincent J-L. Timing of tracheotomy in ICU patients: a systematic review of randomized controlled trials. Crit Care. 2015;19(1). 10.1186/s13054-015-1138-8 PMC4669624 26635016

[40] Johnson AEW, Pollard TJ, Shen L, Lehman LH, Feng M, Ghassemi M, et al. MIMIC-III, a freely accessible critical care database. Sci Data. 2016;3(1). doi: 10.1038/sdata.2016.35 27219127 PMC4878278

[41] Luo W, Phung D, Tran T, Gupta S, Rana S, Karmakar C, et al. Guidelines for Developing and Reporting Machine Learning Predictive Models in Biomedical Research: A Multidisciplinary View. J Med Internet Res. 2016;18(12):e323. doi: 10.2196/jmir.5870 27986644 PMC5238707

[42] Johnson AE, Kramer AA, Clifford GD. A new severity of illness scale using a subset of Acute Physiology And Chronic Health Evaluation data elements shows comparable predictive accuracy. Crit Care Med. 2013;41(7):1711–8. doi: 10.1097/CCM.0b013e31828a24fe 23660729

[43] Vincent JL, de Mendonça A, Cantraine F, Moreno R, Takala J, Suter PM, et al. Use of the SOFA score to assess the incidence of organ dysfunction/failure in intensive care units: results of a multicenter, prospective study. Working group on “sepsis-related problems” of the European Society of Intensive Care Medicine. Crit Care Med. 1998;26(11):1793–800. doi: 10.1097/00003246-199811000-00016 9824069

[44] Le Gall JR, Lemeshow S, Saulnier F. A new Simplified Acute Physiology Score (SAPS II) based on a European/North American multicenter study. JAMA. 1993;270(24):2957–63. doi: 10.1001/jama.270.24.2957 8254858

[45] Le Gall JR, Loirat P, Alperovitch A, Glaser P, Granthil C, Mathieu D, et al. A simplified acute physiology score for ICU patients. Crit Care Med. 1984;12(11):975–7. doi: 10.1097/00003246-198411000-00012 6499483

[46] Knaus WA, Wagner DP, Draper EA, Zimmerman JE, Bergner M, Bastos PG, et al. The APACHE III prognostic system. Risk prediction of hospital mortality for critically ill hospitalized adults. Chest. 1991;100(6):1619–36. doi: 10.1378/chest.100.6.1619 1959406

[47] Le Gall JR, Klar J, Lemeshow S, Saulnier F, Alberti C, Artigas A, et al. The Logistic Organ Dysfunction system. A new way to assess organ dysfunction in the intensive care unit. ICU Scoring Group. JAMA. 1996;276(10):802–10. doi: 10.1001/jama.276.10.802 8769590

[48] Elixhauser A, Steiner C, Harris DR, Coffey RM. Comorbidity Measures for Use with Administrative Data. Med Care. 1998;36(1):8–27. doi: 10.1097/00005650-199801000-00004 9431328

[49] Lundberg SM, Erion G, Chen H, DeGrave A, Prutkin JM, Nair B, et al. Explainable AI for Trees: From Local Explanations to Global Understanding [Internet]. 2019. Available from: http://arxiv.org/abs/1905.04610 10.1038/s42256-019-0138-9 PMC7326367 32607472

[50] Udeh CI, Hadder B, Udeh BL. Validation and Extension of the Prolonged Mechanical Ventilation Prognostic Model (ProVent) Score for Predicting 1-Year Mortality after Prolonged Mechanical Ventilation. Ann Am Thorac Soc. 2015;12(12):1845–51. doi: 10.1513/AnnalsATS.201504-200OC 26418231

[51] Python Core Team. Python [Internet]. Python: A dynamic, open source programming language. Python Software Foundation. [cited 2020 Sep 9]. Available from: https://www.python.org/

[52] Ferrand E, Robert R, Ingrand P, Lemaire F, French LATAREA Group. Withholding and withdrawal of life support in intensive-care units in France: a prospective survey. French LATAREA Group. Lancet Lond Engl. 2001;357(9249):9–14. doi: 10.1016/s0140-6736(00)03564-9 11197395

[53] Sprung CL, Cohen SL, Sjokvist P, Baras M, Bulow HH, Hovilehto S, et al. End-of-life practices in European intensive care units: the Ethicus Study. JAMA. 2003;290(6):790–7. doi: 10.1001/jama.290.6.790 12915432

[54] Bittner EA, Schmidt UH. The ventilator liberation process: update on technique, timing, and termination of tracheostomy. Respir Care. 2012;57(10):1626–34. doi: 10.4187/respcare.01914 23013900

[55] Lamas DJ, Owens RL, Nace RN, Massaro AF, Pertsch NJ, Gass J, et al. Opening the Door: The Experience of Chronic Critical Illness in a Long-Term Acute Care Hospital. Crit Care Med. 2017;45(4):e357–62. doi: 10.1097/CCM.0000000000002094 27632675

[56] Sinuff T, Adhikari NKJ, Cook DJ, Schünemann HJ, Griffith LE, Rocker G, et al. Mortality predictions in the intensive care unit: Comparing physicians with scoring systems. Crit Care Med. 2006;34(3):878–85. doi: 10.1097/01.CCM.0000201881.58644.41 16505667

[57] Fried TR, Bradley EH, Towle VR, Allore H. Understanding the treatment preferences of seriously ill patients. N Engl J Med. 2002;346(14):1061–6. doi: 10.1056/NEJMsa012528 11932474

[58] Marafino BJ, Park M, Davies JM, Thombley R, Luft HS, Sing DC, et al. Validation of Prediction Models for Critical Care Outcomes Using Natural Language Processing of Electronic Health Record Data. JAMA Netw Open. 2018;1(8):e185097. doi: 10.1001/jamanetworkopen.2018.5097 30646310 PMC6324323

[59] Pirracchio R, Petersen ML, Carone M, Rigon MR, Chevret S, van der Laan MJ. Mortality prediction in intensive care units with the Super ICU Learner Algorithm (SICULA): a population-based study. Lancet Respir Med. 2015;3(1):42–52. doi: 10.1016/S2213-2600(14)70239-5 25466337 PMC4321691

[60] Dybowski R, Gant V, Weller P, Chang R. Prediction of outcome in critically ill patients using artificial neural network synthesised by genetic algorithm. The Lancet. 1996;347(9009):1146–50. doi: 10.1016/s0140-6736(96)90609-1 8609749

[61] Kim SY, Kim S, Cho J, Kim YS, Sol IS, Sung Y, et al. A deep learning model for real-time mortality prediction in critically ill children. Crit Care Lond Engl. 2019;23(1):279. doi: 10.1186/s13054-019-2561-z 31412949 PMC6694497

