# Supplementary figures and images for: Deep learning to predict long-term mortality in patients requiring 7 days of mechanical ventilation
Source: PLoS One. 2021 Jun 29;16(6):e0253443. doi: 10.1371/journal.pone.0253443 (PMC8241081; doi:10.1371/journal.pone.0253443)

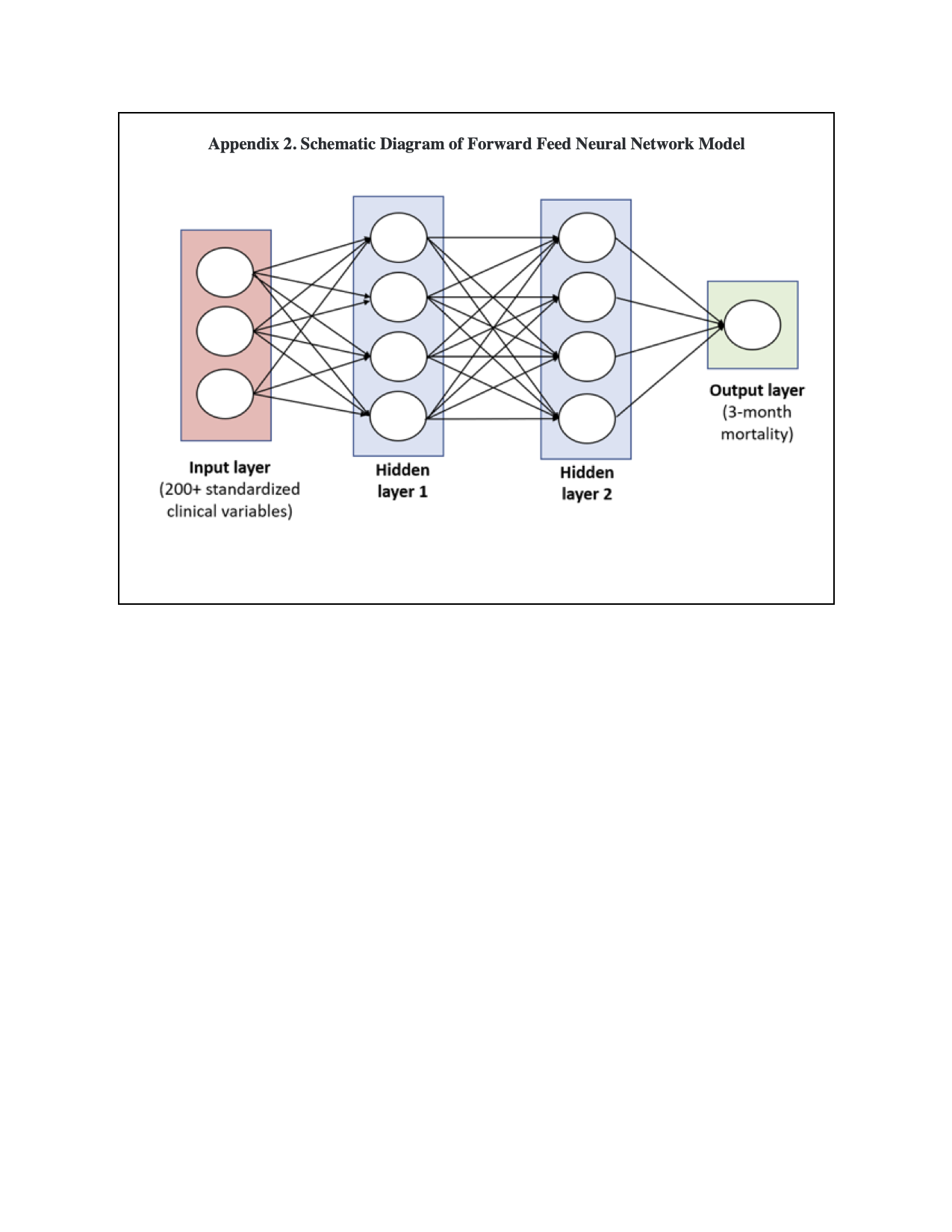

Supplement: S1 Fig — (TIF) [file pone.0253443.s001.tif]

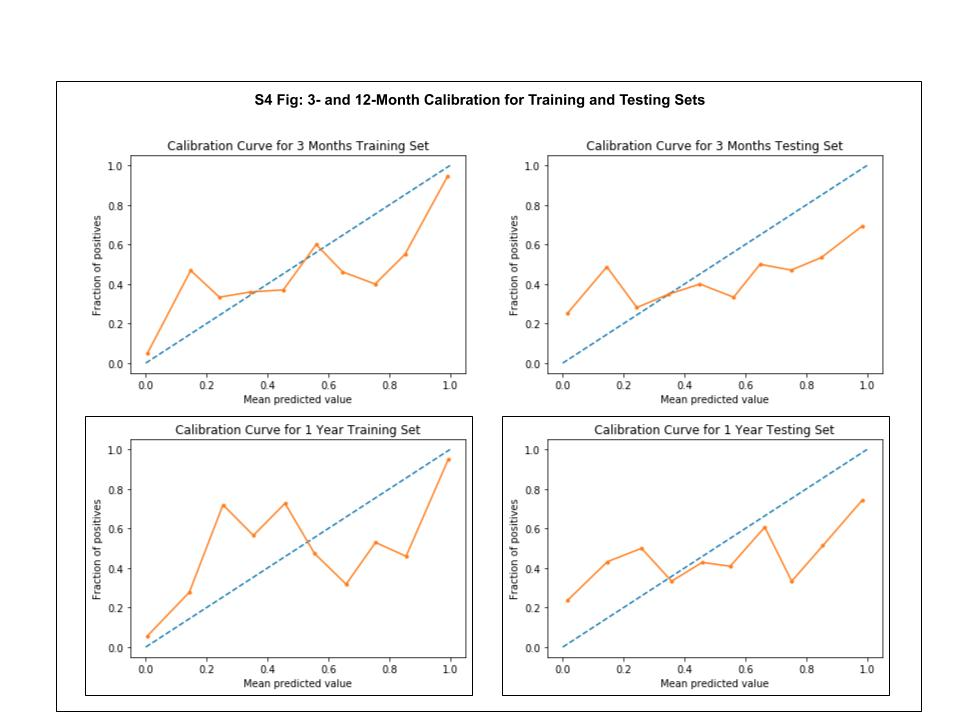

Supplement: S2 Fig — (TIF) [file pone.0253443.s002.tif]

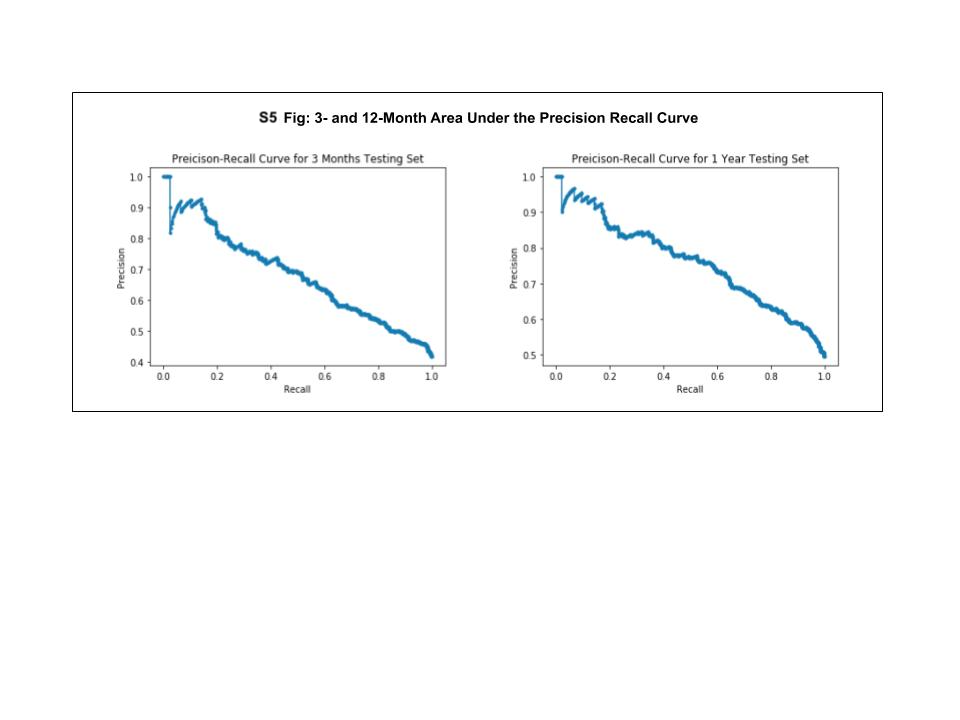

Supplement: S3 Fig — (TIF) [file pone.0253443.s003.tif]

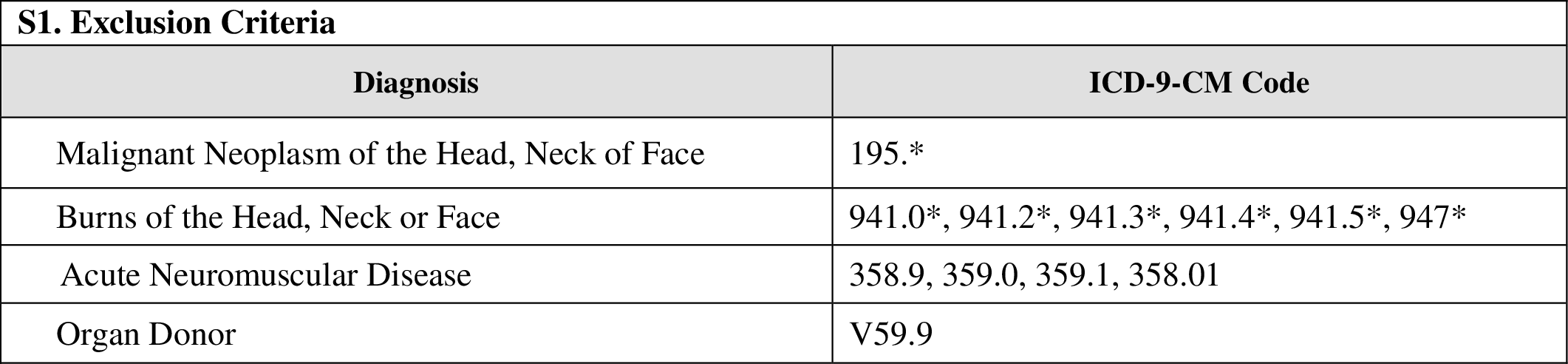

Supplement: S1 Table — (TIF) [file pone.0253443.s004.tif]

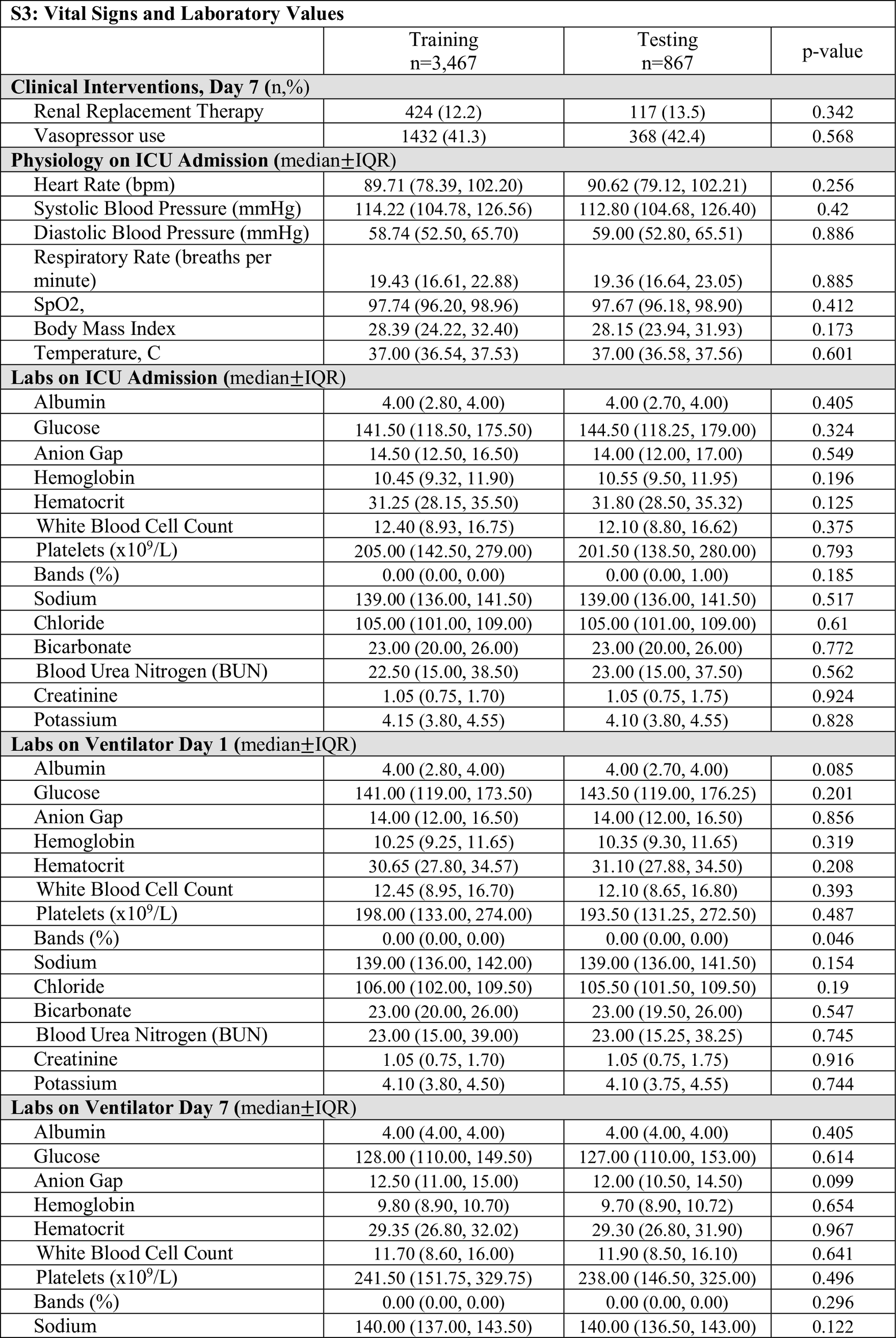

Supplement: S2 Table — (TIF) [file pone.0253443.s005.tif]

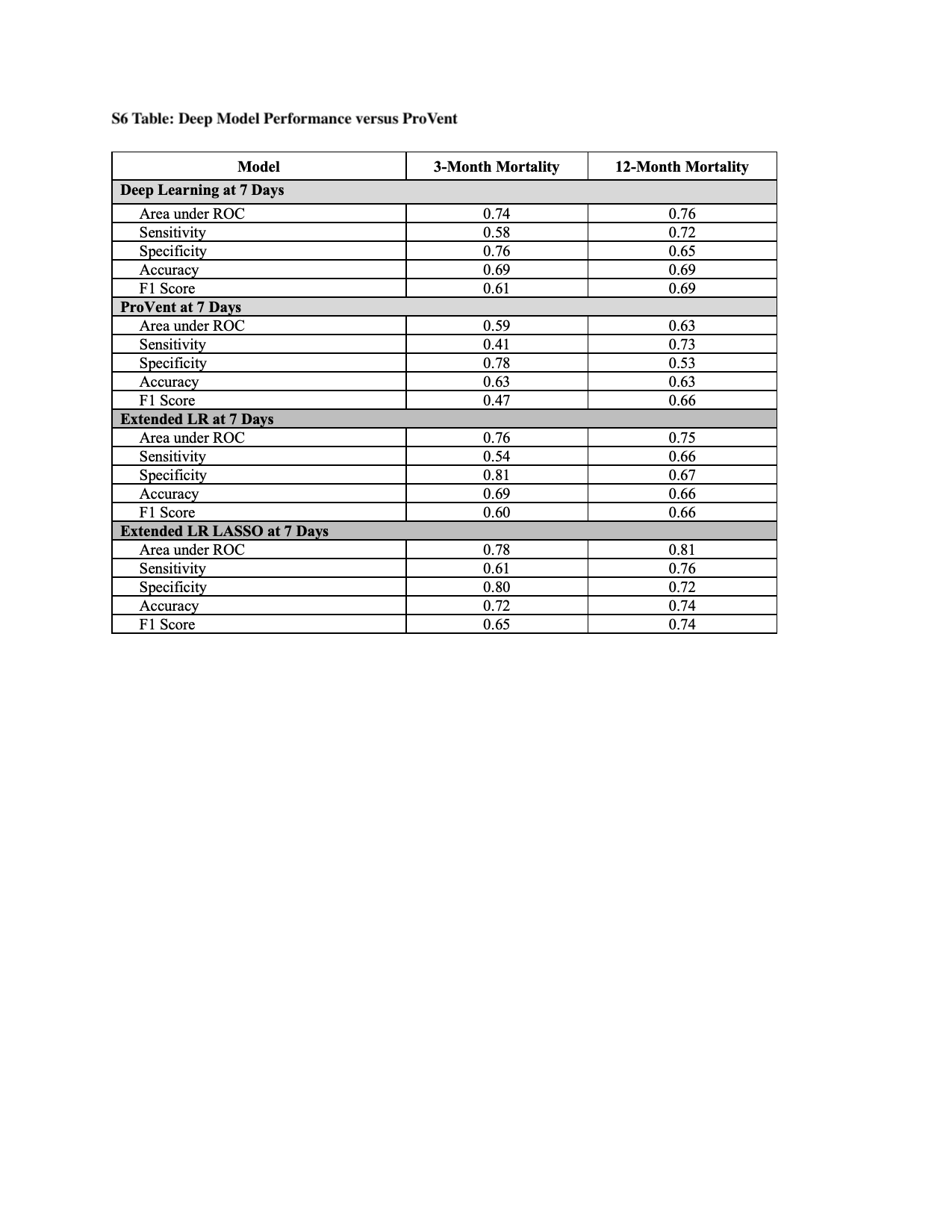

Supplement: S3 Table — (TIF) [file pone.0253443.s006.tif]
