# Supplementary material for: Deep learning to predict long-term mortality in patients requiring 7 days of mechanical ventilation
Source: PLoS One. 2021 Jun 29;16(6):e0253443. doi: 10.1371/journal.pone.0253443 (PMC8241081; doi:10.1371/journal.pone.0253443)
Supplement: Attachment — Submitted filename: Response to Reviewers.docx [file pone.0253443.s008.docx]

Dear Editors,

We appreciate your careful consideration of the first revision of our manuscript. As requested, we have provided a detailed response to the reviewers’ outstanding concerns.

Please do not hesitate to reach out with additional questions or concerns.

Sincerely,

Naomi George

**Reviewer Comment and Author Response:**

Reviewer #2: "The authors have resolved most of my concerns. I have one final comment which if included might strengthen the paper: The authors mention that they trained a multivariate regression model with the same number of variables available to the deep learning model. I do not see the performance of this model reported anywhere in the manuscript. It might be helpful to the reader if the results of a logistic regression model constructed using the same variables as the deep learning model is reported."

Response: Thank you for your comment. The reviewer correctly notes that the reporting in one portion of the methods section should be clearer. To remedy this, and to ensure that the results are consistent with the reported methods, we ran two additional models using logistic regression techniques that utilization all 250 variables available to the neural network. The methods section and results section of the manuscript have been further clarified in the revised draft, and the discussion has been updated to reflect these latest findings. Results for the additional logistic regression models are included as supplemental figure 6 (**S6 FIG)**.
